# Real-time two-dimensional shear wave ultrasound elastography of the liver is a reliable predictor of clinical outcomes and the presence of esophageal varices in patients with compensated liver cirrhosis

**DOI:** 10.3325/cmj.2015.56.470

**Published:** 2015-10

**Authors:** Ivica Grgurević, Tomislav Bokun, Sanda Mustapić, Vladimir Trkulja, Renata Heinzl, Marko Banić, Željko Puljiz, Boris Lukšić, Milan Kujundžić

**Affiliations:** 1 Department of Gastroenterology, University Hospital Dubrava, University of Zagreb School of Medicine and Faculty of Pharmacy and Biochemistry, Zagreb, Croatia; 2 Department of Pharmacology, University of Zagreb School of Medicine, Zagreb, Croatia; 3 Department of Pathology and Cytology, University Hospital Dubrava, Zagreb, Croatia; 4 Department of Gastroenterology, University Hospital Center Split, University of Split School of Medicine, Split, Croatia; 5 Department of Infectious Diseases, University Hospital Center Split, University of Split School of Medicine, Split, Croatia; 6 University of Rijeka School of Medicine, Rijeka, Croatia

## Abstract

**Aim:**

Primary: to evaluate predictivity of liver stiffness (LS), spleen stiffness (SS), and their ratio assessed by real-time 2D shear wave elastography (RT-2D-SWE) for adverse outcomes (hepatic decompensation, hepatocellular carcinoma or death; “event”) in compensated liver cirrhosis (LC) patients. Secondary: to evaluate ability of these measures to discriminate between cirrhotic patients with/without esophageal varices (EV).

**Methods:**

Predictivity of LS, SS, and LS/SS was assessed in a retrospectively analyzed cohort of compensated LC patients (follow-up cohort) and through comparison with incident patients with decompensated cirrhosis (DC) (cross-sectional cohort). Both cohorts were used to evaluate diagnostic properties regarding EV.

**Results:**

In the follow-up cohort (n = 44) 18 patients (40.9%) experienced an “event” over a median period of 28 months. LS≥21.5 kPa at baseline was independently associated with 3.4-fold (95% confidence interval [CI] 1.16-10.4, *P* = 0.026) higher risk of event. Association between SS and outcomes was weaker (*P* = 0.056), while there was no association between LS/SS ratio and outcomes. Patients with DC (n = 43) had higher LS (35.3 vs 18.3 kPa, adjusted difference 65%, 95% CI 43%-90%; *P* < 0.001) than compensated patients at baseline. Adjusted odds of EV increased by 13% (95% CI 7.0%-20.0%; *P* < 0.001) with 1 kPa increase in LS. At cut-offs of 19.7 and 30.3 kPa, LS and SS had 90% and 86.6% negative predictive value, respectively, to exclude EV in compensated patients.

**Conclusion:**

This is the first evaluation of RT-2D-SWE as a prognostic tool in LC. Although preliminary and gathered in a limited sample, our data emphasize the potential of LS to be a reliable predictor of clinical outcomes and the presence of EV in LC patients.

Over the last decade non-invasive methods have increasingly replaced liver biopsy (LB) for the purpose of determination of the stage of liver fibrosis (LF) (1). Among different diagnostic approaches, ultrasound (US) based transient elastography (TE) has been widely accepted by the hepatologists due to its simplicity and reliability, with a huge amount of data to support its use in different etiologies of liver diseases (2). On the other hand, TE has limitations due to the fact that it does not allow two-dimensional (2D) imaging of the investigated structures, cannot be applied in patients with ascites, and investigation of the spleen can be performed only when proper spot has been chosen by conventional ultrasound. These limitations have been overcome by new US devices in which quantitative elastography module has been integrated into conventional abdominal probes (encompassing acoustic radiation force impulse imaging [ARFI] and real-time 2D-shear wave elastography [RT-2D-SWE]), making it possible to perform gray-scale, Doppler, and elastographic investigations at the same time, in the same patient, with the same US probe (3). These elastographic methods have also been tested in different patient populations, mainly with chronic viral hepatitis, and the results were comparable to TE (4,5). Apart from being used to determine the stage of liver fibrosis, TE has been shown to be able to differentiate between the patients with and without clinically significant portal hypertension (especially if combined with other parameters such as platelet counts or spleen size), esophageal varices (EV), and as a reliable prognostic indicator of liver decompensation and death even independent of the history of treatment in patients with viral hepatitis C (6-8). Although most of these outcomes have been related to the liver stiffness (LS) as assessed by TE, spleen stiffness (SS) has recently attracted attention as well. SS correlates even better with portal hypertension and therefore might also predict the risk of hepatic decompensation and death (9,10). Most of these data have been collected by TE, whereas RT-2D-SWE, to the best of our knowledge, has not been evaluated in this respect. The primary aim of this proof-of-concept study was to evaluate whether RT-2D-SWE might be used as a prognostic tool to predict adverse outcomes in patients with compensated cirrhosis by assessing LS, SS, and their ratio. Considering the ability of the method to evaluate LS even in the presence of ascites, we defined two secondary objectives: first, to compare elastographic indicators in compensated liver cirrhosis (LC) patients to a concurrent group of incident patients with decompensated disease, and second, to assess whether elastographic indicators discriminated between the presence and absence of EV.

## Patients and methods

### Design and ethics

**Figure 1:**
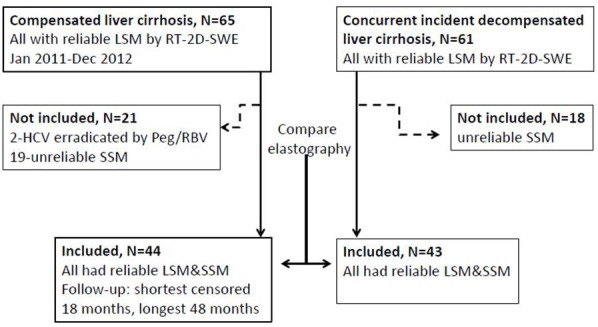
Study outline. LSM – liver stiffness measurement; SSM – spleen stiffness measurement; Peg/RBV – pegylated interferon and ribavirin; RT-2D-SWE – real-time 2D Shear wave elastography.

The present retrospective analysis of a single tertiary-care center (University Hospital Dubrava) database identified two concurrent cohorts of LC patients with reliably assessed LS by RT-2D-SWE during the period from January 2011 to December 2012 (Figure 1): a cohort of compensated LC patients used to assess the predictive value of elastographic data for a subsequent occurrence of death, hepatocellular carcinoma (HCC), or decompensation (a follow-up cohort); and a cohort of patients with incident cirrhosis decompensation used to assesses elastographic indices at decompensation (a cross-sectional cohort) through a comparison to a compensated LC at baseline. If increased organ stiffness turned out to pose a risk for adverse outcomes, decompensated patients would be expected to have higher organ stiffness than compensated ones, which might be considered as a confirmation of the primary finding. Both cohorts were used to assess discriminative properties of elastographic indices in respect to presence/absence of EV. The study was approved by University Hospital Dubrava Ethics Committee. Informed consent was obtained from each participant before any invasive procedure.

### Follow-up cohort (compensated LC)

*Patients*. We included the patients meeting the following criteria: a) biopsy-proven compensated LC irrespective of etiology with diagnostic work-up data sufficient to determine the “model for end-stage liver disease” (MELD) score (11) and “albumin-bilirubin” (ALBI) score (12). To avoid bias arising from a possible effect of an on-going treatment on a long-term outcome, patients with successfully eradicated hepatitis B or C were not included, and the subset of “chronic viral hepatitis” patients actually referred to patients who failed treatment by interferon-based regimens (hepatitis C) or those with hepatitis B treated by nucleoside/-tide analogues, with suppressed HBV-DNA (below threshold of detection), but with persistently positive HBsAg. Patients with primary biliary cirrhosis were treated by ursodesoxicholic acid; patients with non-alcoholic fatty liver disease (NAFLD) were advised on dietary regimens, weight loss, and correction of hypertension, glucose intolerance/diabetes, and hyperlipidemia. Patients with alcoholic cirrhosis (ALC) were advised to abstain from drinking. Therefore, it might be stated that the included patients suffered from an ongoing low-intensity insult to the liver (HBV, HCV, PBC, NAFLD, alcohol). Some of the patients with ALC stated that they had completely abstained, although this is not commonly seen and was difficult to confirm.; b) technically adequately assessed elastographic indices (LS and SS); c) not suffering from conditions known to confound the effect of cirrhosis on elastographic indices: liver congestion due to the right-sided heart failure or pulmonary hypertension, or cholestasis (defined by dilation of bile ducts on ultrasound imaging), or having alanine aminotransferase (ALT)>5 × upper limit of normal.

*Elastographic assessment*. Elastographic measurements were performed on Aixplorer® Ultrasound system, SupersonicImagine, Aix-en-Provence, France, at the Ultrasound unit of the Department of Gastroenterology, by two experienced ultrasonographers (each having performed >300 RT-2D-SWE examinations). All examinations were performed after overnight fasting in supine position with right hand maximally abducted. We used intercostal approach over the anterolateral part of the right liver lobe to obtain real-time elastographic images of the chosen liver area free of artifacts. Elastographic measurements were then performed during 3-4 seconds period of apnea in exhalation. The elastograms were considered representative and reliable only if they fulfilled the following quality criteria: 1) more than two thirds of the elastographic map had to be homogenously colored or have gradual color transition; 2) artifacts (spots/pixelization/lack of signal) occupying less than one third of the elastographic map, and 3) no sharp transition from soft (blue) to hard (red) elastographic areas (13). The Q-box was then placed within the homogenously colored area with proximal edge at least 1.5 cm below the liver capsule and the stiffness measurements expressed in kilopascals (kPa) were recorded. Five measurements per patient were taken from defined anatomic position, after which average (AVG) value and standard deviation (SD) were calculated and only patients with coefficient of variability (CV = SD/AVG)<0.3 for obtained LS values were included in further analysis (14). SS measurements were performed in supine patients, during inbreathing, through the left intercostal spaces, applying the same quality criteria as stated for LS measurements. We chose to obtain elastographic measurements during inbreathing because this approach provided better visualization of the spleen with fewer artifacts. Stiffness ratio index, SRI = (LS/SS) × 10, was also determined (13).

*Clinical and laboratory assessment, outcomes, and case ascertainment*. A standard comprehensive work-up at the time of diagnosis (“baseline”) and elastographic assessment was implemented. EV were classified as “none” [0], small [1], or large/bleeding [2] (15). Also, MELD (11) and ALBI [(log10 **BI**lirubin ×0.66) + (**AL**bumin × - 0.085)] scores were determined (12). Data were reviewed for occurrence of adverse outcomes: death, HCC (based on typical radiological hallmarks or liver biopsy in line with the international guidelines) (16), or any sign of decompensation (jaundice, variceal bleeding, encephalopathy, ascites [in line with the International Ascites Club classification: 0 = no ascites, 1 = small ascites visible only by US, 2 = clinically visible ascites, 3 = large ascites with marked abdominal distension]), whichever occurred first (“event”) (17). Patients not experiencing an event by the date of data review were contacted (if >2 months had elapsed since the last visit), and if lack of an event was confirmed, they were considered “censored.” The primary outcome of interest was time (in months) since diagnosis of cirrhosis and elastographic assessment till the event or end of follow-up (date of data review).

*Sample size and power consideration*. As the study was conceived as exploratory one based on retrospective database review, we did not pre-plan the sample size. We reasoned, however, that a sample of 45 patients would provide reasonable grounds for a proof-of-the concept study: assuming an event rate between 35% and 45% and, based on experience, standard deviation of the elastographic indices in the range between 5 and 20 kPa (RSD between 25% and 100% of the typical kPa readings in compensated LC patients), such a sample would attain between 75% and 98% power at a two-sided 0.05 alpha level to detect a regression coefficient of 0.1397 (hazard ratio, HR = 1.15) with one unit change in an elastographic indicator.

*Data analysis*. Predictive value of elastographic parameters (LS, SS, and SRI) was estimated in univariate and multivariate models. Since the sample was small, multivariate models focused on major known adverse outcome predictors, ie, intended to test predefined hypotheses about elastographic parameters while accounting for major confounding. Considered were demographics, clinical and laboratory measures, and MELD and ALBI scores. Elastographic indicators were first treated as continuous variables, and association with the instantaneous risk of event was estimated using proportional hazard regression. Since the shortest censored period was 18 months, a logistic model was fit to occurrence of an event (yes/no) with the elastographic indicator and time as effects, and a cut-off value of the elastographic indicator at which the estimated probability (“cumulative risk”) of an event over the first 18 months attained 51% was determined. Participants were dichotomized based on this cut-off and proportional hazard regression was repeated with elastographic indicators categorized as “high” (above the cut-off) or “low” (below the cut-off). We conducted several analyses to avoid overparametrization and aliasing in multivariate models. Since at least two known adverse outcome predictors (MELD, ALBI) and 3 elastographic indicators (LS, SS, SRI) were to be considered, we performed principal components analysis (varimax rotation, extraction if eingenvalue >1.0) to evaluate pairwise correlations and “factoring” among them. Having in mind the shown association between SS and portal hypertension (9), and a pathophysiological link between portal hypertension and EV, we evaluated the relationship between elastographic indicators and presence of EV at baseline using two-step cluster analysis and mediation analysis.

### Cross-sectional cohort (patients with incident decompensation)

*Patients*. We included patients with decompensated LC, irrespective of etiology, diagnosed on the basis of typical clinical criteria, presenting with at least one of the following decompensating events: encephalopathy, jaundice, bleeding EV, or ascites (classified as described). Liver biopsy results were available in some patients in whom it had been performed during previous diagnostic work-up, whereas in majority the diagnosis of cirrhosis relied upon clinical criteria (all had history of liver disease accompanied by hemato-biochemical, ultrasonographic, and endoscopic alterations typical for cirrhosis).

*Clinical and laboratory assessment and outcomes*. A standard comprehensive work-up was performed at diagnosis and the same elastographic indicators (meeting the same quality standards) as described for compensated patients were assessed.

*Sample size and power considerations*. As for the prospective cohort, and considering the nature of the study, we did not pre-plan the sample size. We reasoned, however, that a comparable number of patients (around 45, ie, 90 in total) would provide reasonable grounds for a comparison between the two cohorts: for a simultaneous (multivariate) comparison between 2 groups on 3 outcomes, such a sample would provide around 98% power to detect an overall difference defined as an effect size of 0.25 (small) at two-sided 0.05 alpha level. Such a sample would also provide around 84% power to detect an odds ratio of 2.0 at a two-sided 0.05 alpha level in a logistic regression with an increase in the continuous independent variable for one standard deviation and with the assumed baseline event rate between 50% and 60% (expected proportion of patients with EV).

### Joint analysis of data from the follow-up and cross-sectional cohorts

To compare elastography findings between compensated and incident decompensated LC patients, closely related outcomes (LS, SS, and SRI) were analyzed using multivariate multiple regression with adjustment for age and underlying disease. To assess discriminative properties of elastographic indices in respect to EV, logistic regression and receiver operating characteristics (ROC) analysis were performed. Cut-off values with optimum relationship between sensitivity and specificity were determined and positive and negative predictive values were calculated. SAS for Windows 9.3.1 (SAS Inc., Cary, NC) software was used. All tests were performed at the two-sided 0.050 alpha level.

## Results

### Follow-up cohort

*Patient characteristics.* A total of 44 patients were included (36 men, all Child-Pugh A) and were followed-up for a minimum of 18 and a maximum of 48 months (if censored) (Figure 1). The most prevalent underlying cause of LC was alcohol abuse. Baseline patient characteristics are summarized in Table 1. Overall, 18 (40.9%) patients died (n = 8, all but one subsequently to decompensation) or experienced decompensation (n = 17) during the observed period (Table 1).

**Table 1:** Patient characteristics in the “follow-up cohort” (compensated liver cirrhosis). Data are presented as count (%) or median (range)*

	All patients (N = 44)	Death/complications (n = 18)	No events (n = 26)
Men	36 (81.8)	15 (84.4)	21 (80.8)
Age (years)	62.5 (26-79)	60.5 (26-79)	63 (31-73)
Etiology of cirrhosis			
ethanol abuse	20 (45.5)	11 (61.1)	9 (34.6)
hepatitis B	4 (9.1)	2 (11.1)	2 (7.7)
hepatitis C	10 (22.7)	1 (5.6)	9 (34.6)
non-alcoholic fatty liver	6 (13.6)	2 (11.1)	4 (15.4)
primary biliary cirrhosis	3 (6.8)	2 (11.1)	1 (3.9)
hemochromatosis	1 (2.3)	0	1 (3.9)
ascites	0	0	0
Varices			
none	28 (63.6)	7 (38.9)	21 (80.8)
small	13 (29.6)	8 (44.4)	5 (19.2)
large	3 (6.8)	3 (16.7)	0
bilirubin total (μmol/L)	17.8 (5.3-35)	21.4 (8.0-35)	16.2 (5.3-33)
creatinine (μmol/L)	83.5 (57-127)	80.5 (57-127)	84.0 (62.0-106)
albumin (g/L)	42 (25-61)	40.5 (32-58.3)	44.5 (25-61)
INR	1.11 (0.95-1.80)	1.11 (1.02-1.80)	1.11 (0.95-1.50)
ALBI grade numerical	-2.82 (-4.39, -1.12)	-2.61 (-4.16, -1.73)	-2.97 (-4.39, -1.12)
ALBI grade category			
first	27 (61.4)	8 (44.4)	19 (73.1)
second	16 (36.4)	10 (55.6)	6 (23.1)
third	1 (2.2)	0	1 (3.9)
MELD score	9 (6-15)	9.5 (7-15)	8.5 (6-13)
liver stiffness (kPa)	18.3 (9.2-41.1)	23.7 (11.5-41.1)	15.9 (9.2-27.8)
spleen stiffness (kPa)	27.2 (7.8-44.3)	31.2 (20.1-44.3)	26.6 (7.8-34.9)
SRI	6.63 (3.00-17. 3)	7.5 (4.7-12.4)	6.2 (3.0-17.3)
death or complications	18 (40.9)	—	—
deaths	8 (18.2)	—	—
complications	17 (38.6)	—	—

*INR – international normalized ratio; ALBI – albumin-bilirubin grade; MELD – model for end-stage liver disease; SRI – stiffness ratio index = (Liver/Spleen stiffness) × 10.

**Figure 2:**
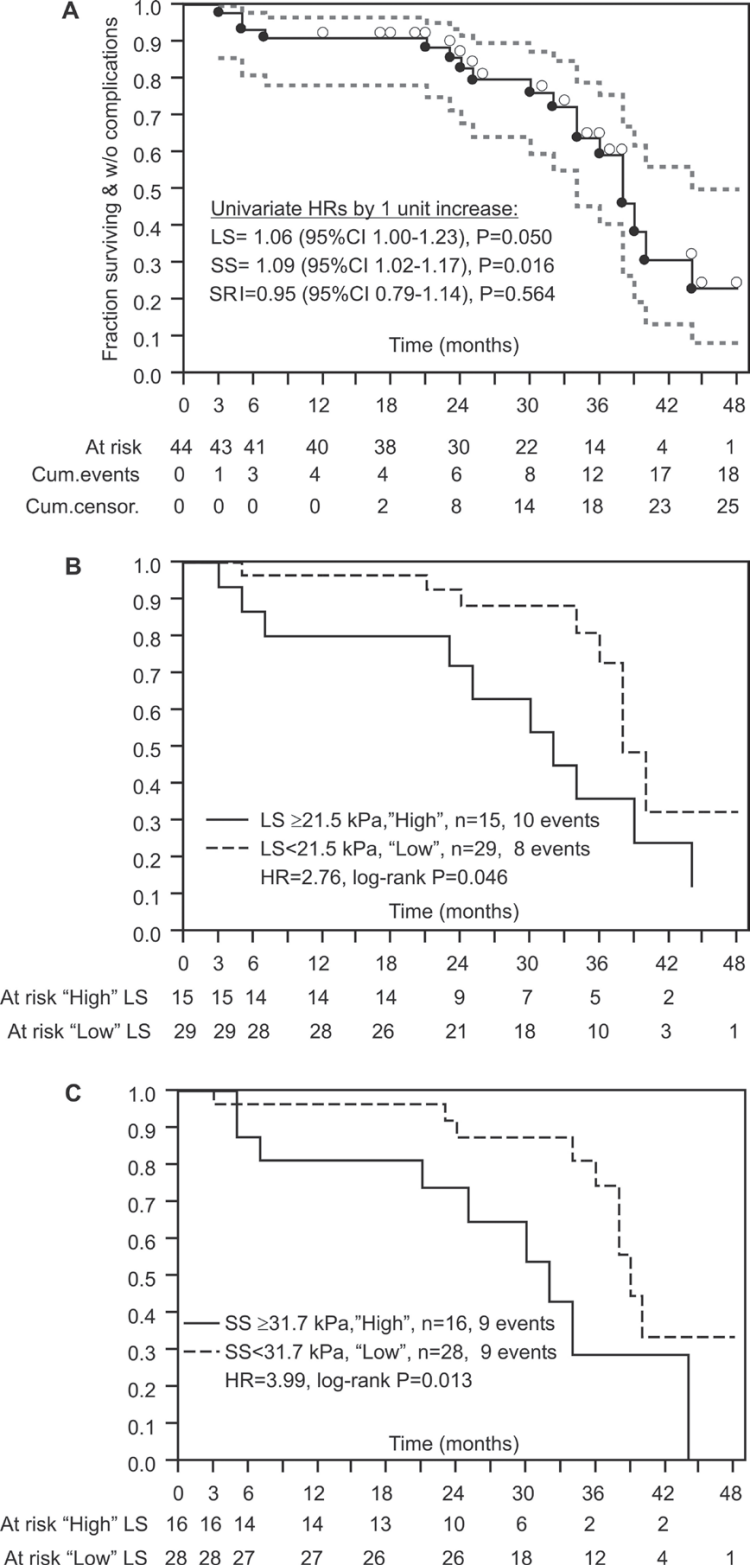
Product-limit curves of time to “event” (death or complication) in a cohort of compensated patients with liver cirrhosis. (**A**) All patients. Depicted are univariate hazard ratios (HRs) for liver stiffness (LS), spleen stiffness (SS), and their ratio index (SRI). Black dots below the curve depict “events,” open circles above the curve depict censorings. (**B**) Data for patients dichotomized by LS≥21.5 kPa (“high”) or <21.5 kPa (“low”). HR is obtained from a Mantel-Haenszel log-rank test. (**C**) Data for patients dichotomized by SS≥31.7 kPa (“high”) or <31.7 kPa (“low”). HR is obtained from a Mantel-Haenszel log-rank test.

*Higher LS and SS predict adverse outcomes in patients with compensated LC.* Figure 2A summarizes occurrence of events (death/complications) over time. Both higher LS and SS (by 1 kPa) were univariately associated with a higher risk of an event (by 6% and 9%, respectively), whereas SRI showed no association. Estimates of a cumulative risk over the first 18 months indicated LS of 21.5 kPa and SS of 31.7 kPa as values at which probability of an event became higher than the probability of no event. In univariate analysis, patients with LS≥21.5 kPa (“high”, n = 15) had a 2.76-fold higher risk of events than those with LS<21.5 kPa (“low”, n = 29) (Figure 2B), whereas patients with SS≥31.7 kPa (“high”, n = 16) had a 3.99-fold higher risk than those with SS<31.7 kPa (“low”, n = 28) (Figure 2C).

**Figure 3:**
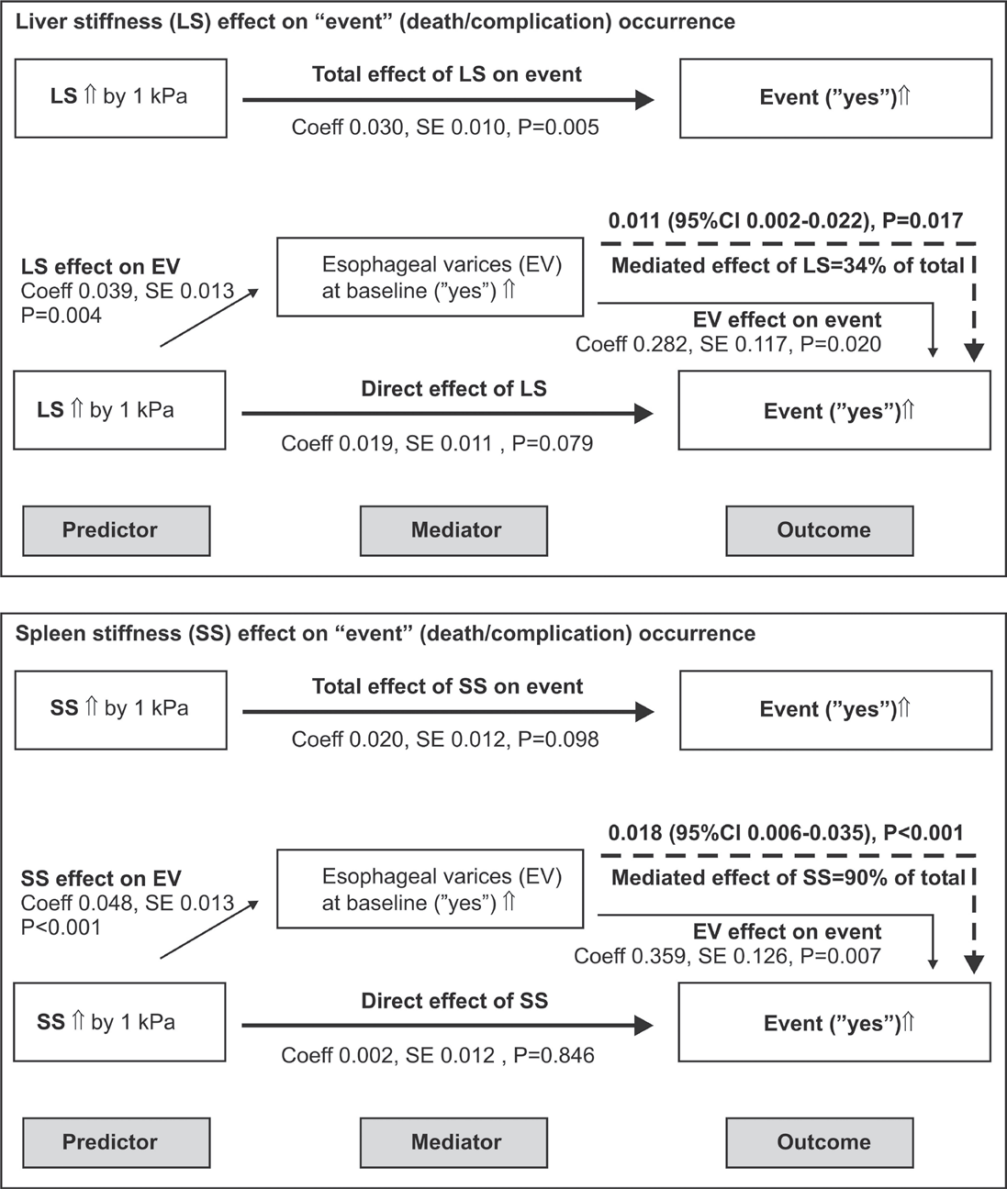
Summary of mediation analysis of the association between liver stiffness (upper panel) or spleen stiffness (lower panel) as predictors, presence of esophageal varices at baseline as a mediator, and subsequent occurrence of death or complication (“event”) (outcome) in compensated patients with liver cirrhosis (N = 44). Shown are “total effects” (coefficients, standard errors, SE) of predictors on the outcome; effects of predictors on the mediator, effects of mediator on the outcome, direct effects of a predictor on the outcome, and mediated effect of the predictor (via mediator variable) on the outcome (thick dashed line). The mediated effect is given with accelerated bias-corrected 95% confidence intervals (CI). Data demonstrate that the “effects” of the liver or spleen stiffness on subsequent occurrence of death or complications are mediated through their effect on presence of esophageal varices at baseline.

In an attempt to rationalize the choice of adjustments in multivariate models, principal components analysis showed that MELD and ALBI scores were closely related and loaded onto the same component, whereas LS and SS were closely related and formed another component (Table 2). Therefore, in order to avoid aliasing of effects, multivariate models were not to simultaneously include MELD and ALBI, or LS and SS, ie, each elastographic indicator was to be assessed separately with adjustment for either MELD or ALBI score. A still considerable correlation between the MELD score and both LS and SS was also observed (Table 2). Two-step cluster analysis based on LS, SS (“high” or “low”), and presence of EV, with good cluster separation (based on Schwartz’s Bayesian Information Criterion) identified two clusters, one (n = 20) in which none of the patients had EV at baseline and all had “low” LS and SS; and the second one (n = 24) in which 67% of patients had EV, 67% had “high” SS, and 63% had “high” LS. Mediation analysis revealed that the association of higher LS and particularly SS with a higher risk of an “event” was largely mediated through their association with the presence of EV at baseline (Figure 3). Hence, the presence of EV was not to be included simultaneously with either LS or SS in multivariate models. Consequently, 4 multivariate proportional hazard regression models were fit to explore the predictive value of LS (either as a continuous or a binary variable, ie, “high” vs “low”), with adjustment of age, ALBI score and serum creatinine (as it is a part of the predictive MELD score); or, with adjustment for age and MELD (instead of ALBI and creatinine). Identical 4 models were fit to explore the predictive value of SS (Table 3). Higher LS was consistently independently associated with a higher risk of event occurrence, except when it was considered as a continuous variable and with adjustment for MELD (Table 3, Model 3 for LS), when the effect was reduced and non-significant, likely due to correlation between LS and MELD (Table 2). The model with the best fit was the one in which, with adjustment for age and MELD score, “high” (≥21.5 kPa) LS was associated with 3.4-fold higher risk of event occurrence (Table 3, Model 4 for LS). Similarly, higher or “high” SS was consistently associated with a higher risk of event occurrence, but when adjusted for MELD score, the effect was reduced and borderline-significant (Table 3, Model 3 and Model 4 for SS). The model with the best fit was the one in which, with adjustment for age and MELD, “high” (≥31.7 kPa) SS was associated with 2.70-fold higher risk of event occurrence, with borderline significance (*P* = 0.056) (Table 3, Model 4 for SS).

**Table 2:** Summary of the principal component analysis*^†^

Correlations	ALBI	MELD	LS	SS
ALBI	1.000	0.582	0.013	0.092
MELD	0.582	1.000	0.399	0.389
LS	0.013	0.399	1.000	0.628
SS	0.092	0.389	0.628	1.000
**Components/loadings**	ALBI-MELD		Elastography	
ALBI	**0.935**		0.084	
MELD	**0.798**		0.418	
LS	0.059		**0.903**	
SS	0.160		**0.875**	

*ALBI – albumin-bilirubin score; MELD – model for end-stage liver disease score; LS – liver stiffness (kPa); SS – spleen stiffness (kPa).

†In the first run, measure of sampling adequacy for “LS/SS ratio” was <0.5 and it was omitted. In the second run, Kaiser-Meyer-Olkin measure of sampling adequacy was 0.555 and all communalities were >0.5. Values in bold indicate loadings >0.5, ie, variables forming the two components.

**Table 3:** Summary of multivariate analysis of predictivity of liver stiffness and spleen stiffness for occurrence of death of decompensation in the “follow-up” cohort of patients with compensated liver cirrhosis*^‡^

	Liver stiffness		Spleen stiffness	
	HR (95% CI)	*P*	HR (95% CI)	*P*
Model 1				
stiffness (by 1 kPa)	1.08 (1.01-1.15)	0.023	1.10 (1.03-1.18)	0.006
age (by 1 year)	1.04 (0.99-1.10)	0.071	1.04 (0.99-1.10)	0.084
ALBI score (by 1 unit)	1.52 (0.81-1.02)	0.204	1.49 (0.80-2.93)	0.213
serum creatinine (by 1 μmol/L)	1.01 (0.98-1.04)	0.405	1.01 (0.98-1.04)	0.397
-2Log Likelihood	91.4		89.0	
AIC	99.4		97.0	
Model 2				
stiffness “high”^†^	5.05 (1.67-16.4)	0.004	4.21 (1.46-12.8)	0.008
age (by 1 year)	1.06 (1.01-1.12)	0.015	1.03 (0.98-1.09)	0.235
ALBI score (by 1 unit)	1.83 (0.93-3.64)	0.079	1.85 (0.91-3.99)	0.088
serum creatinine (by 1 μmol/L)	1.01 (0.98-1.04)	0.479	1.01 (0.98-1.04)	0.481
-2Log Likelihood	88.2		89.5	
AIC	96.3		97.6	
Model 3				
stiffness (by 1 kPa)	1.04 (0.98-1.12)	0.196	1.07 (0.99-1.16)	0.058
age (by 1 year)	1.04 (0.99-1.10)	0.091	1.04 (0.99-1.08)	0.076
MELD score (by 1 unit)	1.23 (0.97-1.57)	0.085	1.21 (0.95-1.52)	0.122
-2Log Likelihood	90.8		88.9	
AIC	96.8		94.9	
Model 4				
stiffness “high”^†^	3.40 (1.16-10.4)	0.026	2.70 (0.96-7.52)	0.056
age (by 1 year)	1.06 (1.01-1.12)	0.029	1.03 (0.99-1.09)	0.166
MELD score (by 1 unit)	1.27 (1.02-1.58)	0.036	1.26 (1.01-1.57)	0.043
-2Log Likelihood	87.5		88.9	
AIC	93.5		94.9	

*HR – hazard ratio; CI – confidence interval; ALBI – albumin-bilirubin score; MELD – model for end-stage liver disease score; AIC – Akaike’s Information Criterion.

†“High” liver stiffness is considered to be ≥21.5 kPa, and “high” spleen stiffness is considered to be ≥31.7 kPa.

‡Proportional hazard assumption was met for all independents in all models (Kolmogorov-type supremum test with 1000 resamplings).

### Cross-sectional cohort and comparison of elastographic indices between compensated and incident decompensated LC patients

**Figure 4:**
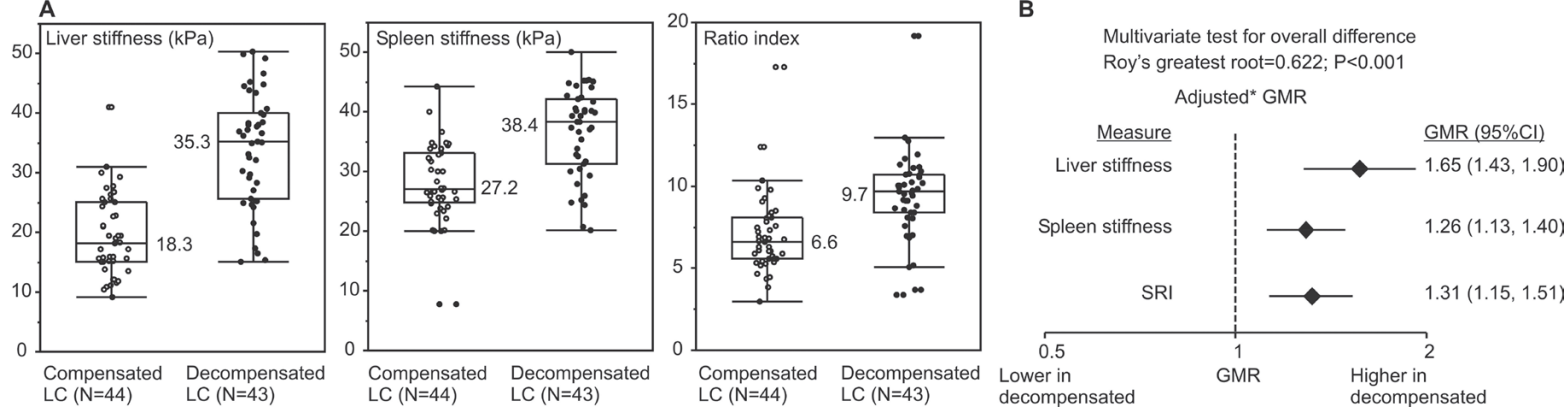
Elastographic parameters in a cohort of patients with compensated and a cohort of incident patients with decompensated liver cirrhosis (LC). (**A**) Individual patient data (open and closed circles) with medians (horizontal bars, numerical values), quartiles (boxes), and inner fences (median ±1.5 [interquartile range]). Values outside fences are outliers. (**B**) Differences in elastographic indicators between incident decompensated and compensated liver cirrhosis patients. The three elastographic measures were analyzed simultaneously in a multivariate multiple regression model with adjustment for age and cirrhosis etiology. Data were ln-transformed due to skewed distribution. The test of overall difference between the two groups is depicted. Adjusted mean differences were exponentiated to obtain adjusted geometric means ratios (GMRs) with 95% confidence intervals. SRI – stiffness ratio index

A total of 43 decompensated LC patients were included. Table 4 summarizes their characteristics. Figure 4A depicts individual and summary elastographic measures in parallel with those obtained from concurrent compensated LC patients (at the start of follow-up). With adjustment for age and cirrhosis etiology (ALC vs other), LS in incident decompensated patients was higher by 65% (95% confidence interval [CI] 43-90), SS by 26% (95% CI 13-40), and SRI by 31% (95% CI 15-51) (Figure 4B). The test for overall difference in elastographic parameters between the two groups was significant (*P* < 0.001) (Figure 4).

**Table 4:** Characteristics of patients with the incident decompensated liver cirrhosis (“cross-sectional cohort”). Data are presented as median (range) or count (%)*

N	43
Men	32 (74.4)
Age (years)	62.8 (38-79)
Etiology of cirrhosis	
ethanol abuse	31 (72.1.5)
hepatitis B	3 (7.0)
hepatitis C	5 (11.6)
non-alcoholic fatty liver	4 (9.3)
Child-Pugh B	25 (58.1)
Child-Pugh C	18 (41.9)
Ascites grade	
0	8 (18.6)
1	18 (41.9)
2	16 (37.2)
3	1 (2.3)
Varices	
none	5 (11.6)
small	22 (51.2)
large	10 (23.3)
bleeding	6 (13.9)
bilirubin total (μmol/L)	39.6 (8.5-323)
creatinine (μmol/L)	87 (56-206)
albumin (g/L)	30 (20-59)
INR	1.50 (1.09-2.70)
ALBI grade numerical	-1.47 (-4.12, -0.32)
MELD score	12 (6-27)
liver stiffness (kPa)	35.3 (15.1-50.3)
spleen stiffness (kPa)	38.4 (20.1-50.1)
SRI	9.68 (3.36-19.2)

*INR – international normalized ratio; ALBI – albumin-bilirubin grade; MELD – model for end-stage liver disease; SRI – stiffness ratio index = (Liver/Spleen stiffness) × 10.

### Elastographic measures discriminate between cirrhotic patients with and without esophageal varices

**Figure 5:**
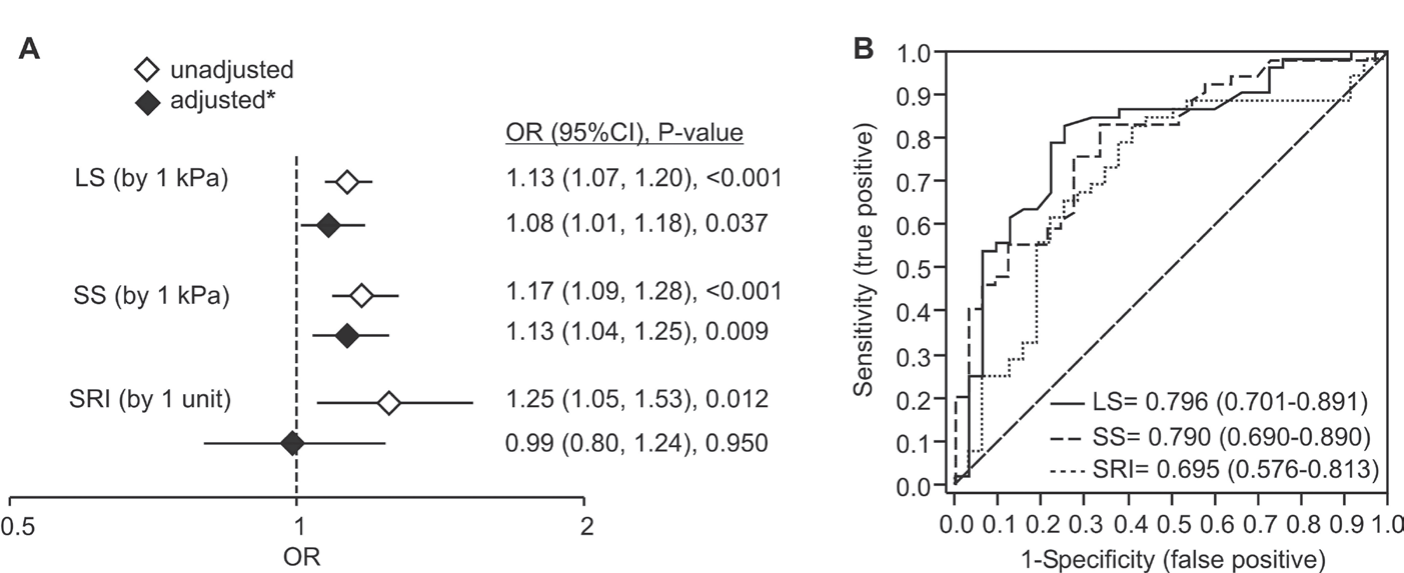
Discriminative properties of liver stiffness (LS), spleen stiffness (SS), and stiffness ratio index (SRI) for the presence of esophageal varices. (**A**) Summary of the logistic regression analysis. Univariate and independent (*adjustment for age, sex, [de]compensation; all *P* < 0.100) odds ratios indicate association between higher LS or SS and higher odds of esophageal varices. Etiology (ethanol abuse vs other) was considered as covariate, as well, but was highly insignificant (*P* > 0.900) and was therefore removed from the model. (**B**) Receiver-operating characteristics (ROC) curves for LS, SS, and SRI. Numerical values are presented with confidence intervals.

Higher LS and SS were both univariately and independently (with adjustment for age, sex, and (de)compensation) associated with higher odds of having EV (Figure 5A). In a univariate test, SRI was also associated with higher odds of EV, but after adjustments no association was apparent (Figure 5A). Area under the ROC curve for LS was in the range of “good discriminative properties” (Figure 5B) and values for SS were similar, whereas discriminative properties of SRI were poor (Figure 5B).

At cut-off values with optimum combination of sensitivity and specificity (19.7 kPa for LS and 30.3 kPa for SS), sensitivity (83.3% and 79.6%, respectively) and specificity (66.3% and 75.8%, respectively) for both LS and SS were moderate (Table 5). With a high prevalence rate (62%) in the present sample this resulted in modest positive and, in particular, negative predictive values (NPV) (Table 5). However, since predictive values largely depend on the event prevalence, present results should be considered with a caution. As depicted in Table 5, using these sensitivity and specificity results and assuming event prevalence of 10% or 20%, NPVs for both LS and SS (at their respective cut-offs) were entirely >90% (95% CIs >90%). At 35% event prevalence (as in the current “prospective cohort”, ie, compensated LC patients), NPV for LS (cut-off 19.7 kPa) was around 90%, whereas for SS (cut-off 30.3 kPa) it fell just below 90% (Table 5).

**Table 5:** Diagnostic performance of liver stiffness and spleen stiffness in detection of esophageal varices in patients with liver cirrhosis

	Liver stiffness	Spleen stiffness
Number of patients	87	87
Event prevalence [n (%)]	54 (62.1)	54 (62.1)
Optimum* cut-off (kPa)	19.7	30.3
Sensitivity (true positive rate) (%)	83.3 (70.7, 92.1)	79.6 (66.5, 89.4)
Specificity (true negative rate) (%)	66.6 (48.2, 82.0)	75.8 (57.7, 88.9)
1-specificity (false positive rate) (%)	33.4 (18.0, 51.8)	24.2 (11.1, 42.3)
1-sensitivity (false negative rate) (%)	16.7 (7.9, 29.3)	20.4 (10.6, 33.5)
Positive predictive value (%)	80.4 (51.0, 89.8)	84.3 (71.4, 92.9)
Negative predictive value (NPV) (%)	71.0 (52.0, 85.8)	69.4 (51.9, 83.6)
Event probability – negative test (%)	29.0 (14.2-48.0)	30.6 (16.4, 48.1)
Likelihood ratio – positive test	2.5 (1.6, 4.3)	3.3 (1.9, 6.3)
Likelihood ratio – negative test	0.25 (0.13, 0.46)	0.27 (0.15, 0.46)
Diagnostic odds ratio	9.7 (3.3, 31.5)	11.8 (3.9, 39.5)
Estimated NPV at 10% prevalence	97.2 (95.6, 98.4)	97.1 (95.6, 98.3)
Estimated NPV at 20% prevalence	94.0 (91.6, 95.8)	93.8 (91.6, 95.6)
Estimated NPV at 35% prevalence (as in compensated)	90.0 (86.7, 91.9)	87.6 (84.5, 90.2)

*Optimum combination of sensitivity and specificity.

## Discussion

Decades ago cirrhotic patients were recognized as a rather heterogenous group with a variable survival and risk for complications following surgery, as depicted through Child-Pugh scoring system (18). Although Child-Pugh system clearly pointed to the dynamic nature of LC, this has been only recently recognized based on results from natural history studies, pathohistological and molecular research. Based on these findings, LC is nowadays considered an evolving entity as opposed to a previous dogma of it being a final and stationary stage of chronic liver disease (19). Accumulation of fibrosis is one of the most prominent hallmarks of chronic liver disease, tightly related to deterioration of liver functions and clinical outcomes, and for these reasons it has been used for staging purposes (20,21). Cirrhosis is characterized by continuous changes at the molecular level, fibrosis accumulation, scar consolidation, architectural distortion, and neovascularization along with development of portal hypertension and reduction in liver function (22). Increasing amount of LF makes the liver stiffer, which can be monitored by external devices based on elastography. Indeed, liver stiffness as measured by TE represents a genuine feature reflecting the amount of liver fibrosis and is a reliable prognostic indicator (23). In a large cohort of patients with chronic hepatitis C, higher LS as assessed by TE was independently associated with a shorter survival and higher rate of liver-related complications (decompensation, death, HCC) regardless of the history of therapy (8). RT-2D-SWE is a newer ultrasound-based elastography method that offers several technical advantages over TE. So far, it has not been evaluated as a tool for assessment of risk of complications in compensated LC patients. The present preliminary study is the first evaluation of RT-2D-SWE in this setting, and furthermore, the first evaluation of the method as a tool for non-invasive diagnosis of EV. The study suffers from limitations inherent to its retrospective design and a relatively small, single-center sample. However, for several reasons we believe that the observations were fairly well protected from different biases: all patients in the prospective (follow-up) cohort underwent a standardized diagnostic work-up at baseline with LC verified by liver biopsy and all were followed-up closely with a standardized procedures of event ascertainment; all patients in both cohorts underwent a standardized and reliable sonographic assessment; and a variety of potentially confounding factors were considered in data analysis. Different etiologies of LC in the present sample could also be viewed as a confounding factor (since “etiology” was not included in the present regression models), however previous studies with TE demonstrated that the association between liver stiffness (fibrosis) and clinical prognosis was not conditional on LC etiology (23). Finally, generalizability of observations from the “prospective cohort” is limited by the fact that it included a subset of selected patients that could be perceived as those with an on-going exposure to an offending agent at a “low-intensity level.” Under these circumstances, the present study strongly points out a high potential of RT-2D-SWE-assessed LS and, less so, SS (but not their ratio) as prognostic and diagnostic tools in two settings in LC patients which deserve further evaluation and confirmation in larger prospective studies. First, in the present carefully built analysis models, higher baseline LS (and less so SS) was independently associated with a higher risk of adverse clinical outcomes (composite of death, HCC, or decompensation). The prognostic value of LS is further supported through the comparison between the compensated and incident decompensated patients – the values in decompensated patients were considerably higher. This observation points to the fact that accumulation and changes in bio-physical properties of fibrous tissue continue to develop within the cirrhotic liver along with evolution of its clinical stages. We also noticed that EV developed at lower values of LS as compared to ascites formation, which is in line with the natural history of LC (24). This is logical since ascites represents a decompensating event of LC that occurs at higher level of portal pressure close to the values at which EV start to bleed (19). The present analysis further suggests that the association between LS (and SS) and adverse clinical outcomes in compensated patients is largely mediated through their association with EV formation and development. This is not surprising since the severity of portal hypertension (with a consequent EV formation and evolution) is the best single predictor of adverse outcomes in LC (9,10,25). Second, the present data demonstrate that LS by RT-2D SWE differentiates between patients with and without EV and emphasize its potential as a diagnostic test for EV. A similar application has been suggested for TE and ARFI based elastography, but diagnostic properties did not seem satisfactory (26). The cut-off values with optimum combination of sensitivity and specificity and the corresponding negative (which are of a particular interest) and positive predictive values in the current sample should be taken with caution due to the sample properties and dependence on event prevalence. However, projections made toward populations with lower expected event prevalence (eg, 10%, 20% or 30%), ie, patients with early cirrhosis who would actually be the target population for a noninvasive screening diagnostic procedure (a high negative predictive value would be desirable) support the view that RT-2D SWE-based LS has a high potential that deserves further investigation.

In conclusion, although preliminary and gathered in a limited sample, the present real-life data for the first time strongly emphasize a high potential of LS assessed by RT-2D-SWE method as a simple and reliable predictor (risk stratification factor) of clinical outcomes in patients with compensated liver cirrhosis, and a noninvasive diagnostic test for esophageal varices in patients with liver cirrhosis. Based on the current results, the prognostic/diagnostic properties of SS appear less promising, but both deserve further evaluation in adequately designed prospective studies.

## References

[1] EASL-ALEH Clinical Practice Guidelines (2015). Non-invasive tests for evaluation of liver disease severity and prognosis.. J Hepatol.

[2] Tapper EB, Afdhal NH (2015). Vibration-controlled transient elastography: a practical approach to the noninvasive assessment of liver fibrosis.. Curr Opin Gastroenterol.

[3] Bamber J, Cosgrove D, Dietrich CF, Fromageau J, Bojunga J, Calliada F (2013). EFSUMB guidelines and recommendations on the clinical use of ultrasound elastography. Part 1: Basic principles and technology.. Ultraschall Med.

[4] Nierhoff J, Chávez Ortiz AA, Herrmann E, Zeuzem S, Friedrich-Rust M (2013). The efficiency of acoustic radiation force impulse imaging for the staging of liver fibrosis: a meta-analysis.. Eur Radiol.

[5] Cassinotto C, Lapuyade B, Mouries A, Hiriart J-B, Vergniol J, Gaye D (2014). Non-invasive assessment of liver fibrosis with impulse elastography: Comparison of Supersonic Shear Imaging with ARFI and FibroScan®.. J Hepatol.

[6] Berzigotti A, Seijo S, Arena U, Abraldes JG, Vizzutti F, García-Pagán JC (2013). Elastography, spleen size, and platelet count identify portal hypertension in patients with compensated cirrhosis.. Gastroenterology.

[7] Augustin S, Millán L, González A, Martell M, Gelabert A, Segarra A (2014). Detection of early portal hypertension with routine data and liver stiffness in patients with asymptomatic liver disease: a prospective study.. J Hepatol.

[8] Vergniol J, Foucher J, Terrebonne E, Bernard P-H, le Bail B, Merrouche W (2011). Noninvasive tests for fibrosis and liver stiffness predict 5-year outcomes of patients with chronic hepatitis C. Gastroenterology.

[9] Colecchia A, Montrone L, Scaioli E, Bacchi-Reggiani ML, Colli A, Casazza G (2012). Measurement of spleen stiffness to evaluate portal hypertension and the presence of esophageal varices in patients with HCV-related cirrhosis.. Gastroenterology.

[10] Colecchia A, Colli A, Casazza G, Mandolesi D, Schiumerini R, Reggiani LB (2014). Spleen stiffness measurement can predict clinical complications in compensated HCV-related cirrhosis: a prospective study.. J Hepatol.

[11] Wiesner R, Edwards E, Freeman R, Harper A, Kim R, Kamath P (2003). Model for end-stage liver disease (MELD) and allocation of donor livers.. Gastroenterology.

[12] Johnson PJ, Berhane S, Kagebayashi C, Satomura S, Teng M, Reeves HL (2015). Assessment of liver function in patients with hepatocellular carcinoma: a new evidence-based approach-the ALBI grade.. J Clin Oncol.

[13] Grgurevic I, Puljiz Z, Brnic D, Bokun T, Heinzl R, Lukic A (2015). Liver and spleen stiffness and their ratio assessed by real-time two dimensional-shear wave elastography in patients with liver fibrosis and cirrhosis due to chronic viral hepatitis.. Eur Radiol.

[14] Reed GF, Lynn F, Meade BD (2002). Use of coefficient of variation in assessing variability of quantitative assays.. Clin Diagn Lab Immunol.

[15] De Franchis R, Pascal JP, Ancona E, Burroughs AK, Henderson M, Fleig W (1992). Definitions, methodology and therapeutic strategies in portal hypertension. A Consensus Development Workshop, Baveno, Lake Maggiore, Italy, April 5 and 6, 1990.. J Hepatol.

[16] (2012). EASL-EORTC clinical practice guidelines: management of hepatocellular carcinoma.. J Hepatol.

[17] (2010). EASL clinical practice guidelines on the management of ascites, spontaneous bacterial peritonitis, and hepatorenal syndrome in cirrhosis.. J Hepatol.

[18] Pugh R, Murray-Lyon I, Dawson J (1973). Transection of the esophagus for bleeding esophageal varices.. Br J Surg.

[19] Garcia-Tsao G, Friedman S, Iredale J, Pinzani M (2010). Now there are many (stages) where before there was one: In search of a pathophysiological classification of cirrhosis.. Hepatology.

[20] Khan MH, Farrell GC, Byth K, Lin R, Weltman M, George J (2000). Which patients with hepatitis C develop liver complications?. Hepatology.

[21] Germani G, Burroughs AK, Dhillon AP (2010). The relationship between liver disease stage and liver fibrosis: a tangled web.. Histopathology.

[22] Hytiroglou P, Snover DC, Alves V, Balabaud C, Bhathal PS, Bioulac-Sage P (2012). Beyond “cirrhosis”: a proposal from the International Liver Pathology Study Group.. Am J Clin Pathol.

[23] Singh S, Fujii LL, Murad MH, Wang Z, Asrani SK, Ehman RL (2013). Liver stiffness is associated with risk of decompensation, liver cancer, and death in patients with chronic liver diseases: a systematic review and meta-analysis. Clin Gastroenterol Hepatol.

[24] D’Amico G, Garcia-Tsao G, Pagliaro L (2006). Natural history and prognostic indicators of survival in cirrhosis: a systematic review of 118 studies.. J Hepatol.

[25] Ripoll C, Groszmann R, Garcia-Tsao G, Grace N, Burroughs A, Planas R (2007). Hepatic venous pressure gradient predicts clinical decompensation in patients with compensated cirrhosis.. Gastroenterology.

[26] Shi K-Q, Fan Y-C, Pan Z-Z, Lin X-F, Liu W-Y, Chen Y-P (2013). Transient elastography: a meta-analysis of diagnostic accuracy in evaluation of portal hypertension in chronic liver disease.. Liver Int.

